# May the Calcific Elements of the Deciduous Teeth Be Appropriated in the Formation of Any Portion of the Permanent Ones?

**Published:** 1873-11

**Authors:** M. S. Dean

**Affiliations:** Chicago.


### ARTICLE II.

### *May the Calcific Elements of the Deciduous Teeth be Appropriated in the Formation of any Portion of the Permanent Ones?*

BY M. S. DEAN, D. D. S., CHICAGO.

Read before the Illinois State Dental Society.

I propose to show that the lime salts of the deciduous teeth may be (are) used again in the formation of parts of the permanent ones, or in the growth of tissues requiring these materials I do not claim to be able to prove this by original researches in chemistry or physiology, but only by natural conclusions from well-established physiological facts.

If we take a comprehensive survey of the general features of this subject, simply in the light of common sense, we shall admire the exquisite skill with which the human body is framed. In it we shall find beauty, strength and compactness harmoniously blended; we shall be struck with amazement at the *economy* which manifests itself, everywhere, in the construction and maintenance of each individual part. In the formation of the osseous system, for instance, we shall see lightness combined with inflexibility and strength; and wherever it becomes necessary to use the bones for fulcrums or as attachments for the muscles, their bulk is increased, while their weight and strength still retain their just proportion to the other parts. In short, they are composed of materials so arranged that the least possible quantity of matter will yield the greatest amount of durability and strength.

Then, again, we shall observe that the *supply* of materials that is furnished for the growth and repair of the organs is used with scrupulous economy. No portion is lost or allowed to go to waste. To guard against this waste of material, the system is traversed by a host of little gleaners—the lymphatics—which gather up from the interstitial parts all the strayed exudations, and carry them into the general circulation; so that nothing which can be assimilated or appropriated by any organ or tissue is permitted to go to waste, or escape from the system as excreted substance, unless there be an excess of these ingredients in the system.

So marvelous do we find the plan of economy, upon which the complex machinery of the various functions of our bodies is carried on, that a cursory examination even of its details must convince the most skeptical that "The hand that made us is divine," and that this divine hand is shown most conspicuously in the ECONOMY of the *forces* and the *materials* employed in the construction and maintenance of the human body.

Now, as a familiar example illustrative of the *economy* with which the system may use the *inorganic* materials introduced in the food, the management and disposition of common salt affords an excellent one. And upon this point I cannot do better than quote the words of Draper, who, I believe, expresses the opinion of physiologists generally upon this subject. He says, : "Oxyde of sodium, or soda, is a uniform ingredient of the bile. The hydrochloric acid of the gastric juice, and soda of the bile, are derived from the same source—common salt—which is either present in the food or purposely added as a condiment. It undergoes decomposition easily, yielding the two products specified, that is, hydrochloric acid and soda, and is readily formed by the union of these two substances. Common salt introduced into the system undergoes decomposition, furnishing hydrochloric acid to the gastric juice and soda to the bile. Considering the large quantity of these secretions produced in a short space of time, it is clear that the drain of common salt must be great—not less than a third of an ounce a day; yet the quantities consumed, at least, are only small. How, then, is this explained? Assuredly, there is no other source from which these bodies can come than the one indicated—the common salt—and yet it seems to be totally inadequate." He says further: "I think this difficulty is rather imaginary than real. Things are so arranged that a limited quantity of salt can produce unlimited quantities of gastric juice and bile; for the former, associated with the food it has digested, scarcely escapes from the pyloric valve before it encounters the bile and pancreatic juices discharging into the duodenum, and through the length of the upper portion of the small intestines these secretions, together with the food they have acted upon, are brought into complete contact. The reproduction of chloride of sodium is, therefore, constantly taking place in intestinal digestion, and it returns back into the system through the absorbents. Again, it undergoes decomposition, its acid reappearing in the gastric juice, and its alkali in the pancreatic juice and bile. By thus using a small amount over and over again, great effects can be produced, and it is then only necessary to restore those small portions that are wasted in carrying out the general scheme."

Here Draper has given a beautiful illustration of the wonderful economy with which the system may use the *inorganic materials*. Were all the elements introduced in the food incapable of further use after having served one purpose in the system, the body would be overloaded by the mass of materials it would have to carry, to such an extent, perhaps, as to nearly deprive the animal of locomotion.

This faculty—if I may use the expression—of using again and again the same identical ingredients—this power of successive decomposition and redecomposition of common salt in the system would seem to solve the mystery which I have been unable, until now, to account for, and that is the thrifty appearance which neat cattle maintain for many months after the external supply of this essential material in nutrition is cut off. Their internal supply is sufficient as long as it is retained in the system. And, on the other hand, the daily escape of a minute portion of this ingredient with the excrements would account also for the subsequent languid and unhealthy condition of these animals when deprived of a new supply for a greater length of time. Both of these facts have become notorious, that is, that cattle do not seem to suffer in health when deprived of salt for several months; but if this deprivation be continued for a long time, say six to twelve months, the appearance of these animals contrasts very unfavorably with other cattle which are fed the same kind and quantity of food, with the addition of salt.

From this general view of the *providence* and *economy* of nature, it is unlikely, *a priori*, that the rich supply of calcific matter taken from the deciduous teeth should be allowed to escape from the system as waste, especially when the same kind of materials is needed for growth or repair in many parts of the body.

Now let us for a moment examine the nature of the materials removed by the absorbents from the deciduous teeth, and see whether they are capable of being re-employed.

In the first place, they are living materials, in one sense at least, although Beale calls all "formed material" dead material. But I shall apply the term *living* material to the ingredients of teeth in which the pulp is not devitalized, to contradistinguish it from the constituents of teeth in which the pulp is dead.

These materials, in deciduous teeth, are not absorbed *on account* of their degeneration; they are not taken away by these vessels *because* they have become disintegrated, and thereby rendered less capable of performing their functions in the tissues or organs of which they are constituent parts, than they were *before* the absorbents seized upon them and dissolved their combinations. But these materials—these teeth—are taken out of the way, because they are a physical obstacle that must be removed, in order to give room for their stronger and more enduring successors.

The removal of these materials is the result of physiological or vital action, and is not a pathological or chemical process. If it were the latter (chemical action) the pulpless or dead teeth might also be removed by the same process. The dead teeth, though they yield as readily to the action of chemical agents as do the living ones, resist effectually and totally the absorbent forces of the papillæ. These materials, then, are living undegenerate matter, taken up by vital or physiological forces, and carried into the circulation, and there can be no reason whatever for supposing they have become unfit pabulum for other tissues. Some may contend that the lime salts taken from these teeth have lost their connection with the organic matter, with which they have been intimately associated, and for this reason cannot be appropriated by the tissues. They may take the ground that the phosphates have become disorganized, so to speak, and that these identical ingredients cannot be again assimilated until they have become re-organized, as it were, in the vegetable form, and in that form enter the system again. This objection to the use of the calcareous elements of the deciduous teeth is, I think, utterly groundless, even if they had lost their intimate union with organic matter; for it is altogether probable that the crude phosphates may form this connection with organic matter *within the system.* But if we grant that this combination cannot be effected internally, it by no means proves that these calcific elements cannot be appropriated by the tissues, for all the authorities I have noticed upon this point agree that the phosphates leave the body in the same form in which they enter it in the vegetable food. If this be the case, the objection to the re-appropriation of these materials, on account of the disintegration of the organic matrix, or their separation from organic matter in which they have been held, is without a shadow of foundation.

That this waste of the lime salts of the deciduous teeth would be contrary to the economy of nature, I think I have already shown; and that it is contrary to other analagous processes in nutrition, I think I shall be able to furnish sufficient evidence.

The opinion of physiologists generally, is by no means adverse to this view. Lionel S. Beale, says:—" In the formation of muscle, for instance, from lifeless nutrient pabulum in the blood, the matter which is to become muscle passes through these different conditions:

" 1. That of soluble nutrient matter, or pabulum.

" 2. Germinal matter, nucleus.

" 3. Imperfectly developed, formed material.

" 4. Fully developed, formed material, muscular contractile tissue.

" 5. Disintegrated formed material, which becomes slowly reduced to a soluble state, and is converted, by oxydation into new substances, some of which pass away, while others in their turn become pabulum for other kinds of germinal matter, such as the white blood corpuscles and the lymph corpuscles."

Here Beale makes the direct statement that some of the disintegrated muscle tissue may be used again as pabulum for germinal matter. And as far as his authority goes—and I believe there is none greater—the question is already settled that materials which have been used in the formation of one tissue may be afterwards appropriated by others. Dunglinson, Carpenter, Dalton, Flint and Paget, as far as their testimony goes, it is indirectly confirmatory of this statement of Beale, though they are more reticent on this point than Draper, who iterates the same sentiment in these words:—" The constitution of the urine proves that the amount of muscular fibrin destroyed in short periods of time is very great. We cannot estimate the hourly consumption at less than 62 grains. Such a waste must demand an equivalent compensation, if the animal mechanism is to be kept unimpaired, and every care is therefore taken to omit no means which may incidentally offer for husbanding the necessary materials. The action of the lymphatics illustrates this principal significantly. Passing through all the soft solids, where exudation of albumen from the blood-vessels can take place, they collect the materials that would otherwise go to waste, and add thereto many of the products arising from disintegration and decay of the soft parts themselves."

In regard to the development and maintenance of the adipose and osseous tissues, he says: "From the phenomena of nutrition generally, we may conclude that there are several sources from which *materials* for these purposes may be derived; a part may be obtained directly from the food; a part may be manufactured or fabricated in the system itself, or *may be taken from some locality therein*, in which it has become redundant or useless, and transferred elsewhere to the point at which it is required."

Again, he says:—"In the adult the sources of bone-earth are two-fold; in part it is derived from the food, and in part from the re-modeling and changes of the bones themselves."

The same author makes the following clinching statement, which, if possible, is still more to the point:—"As to the sources from which phosphate of lime are derived, though doubtless the food offers it in considerable quantity, there are many reasons for inferring that the identical portion which has been removed from one part, is used for the extension of another; and thus we may say that there is a plastic operation continually going forward, a re-modeling, so as to adapt the structure to its new conditions if in a growing animal, or to maintain it in good repair if in an adult."

These statements of Beale and Draper, if worthy of credence, are sufficient analogical evidence to satisfy me that the calcific elements of the deciduous teeth may be appropriated in building up portions of the permanent teeth, or any of the other tissues of the body that require calcareous elements.

So also Liebig—"It is obvious that the phosphoric acid which in consequence of the metamorphosis of tissues is produced in the form of soluble alkaline phosphates must re-enter the circulation of this class of animals (the graminivora.) It is then employed in forming brain and nervous matter, to which it is essential, and also, no doubt, in contributing to the supply of the earthy part of the bones. In the graminivora, therefore, whose food contains so small a portion of phosphates, the organism collects all the soluble phosphates produced by the metamorphosis of tissues, and employs them for the development of the bones and the phosphorised constituents of the brain; the organs of excretion do not separate these salts from the blood."

We have seen from the foregoing—

That the process of nutrition is carried on in an exceedingly prudent and economical manner; that the elements taken from the deciduous teeth by the absorbents are not degenerated materials, nor unfit pabulum for other tissues; that the inorganic substance—common salt—may be used over and over again, subserving an important purpose in nutrition, until it is gradually lost with the excrementitious matters; we have seen that portions of the disintegrated muscles, which are composed almost entirely of inorganic matter, may be converted into pabulum for other tissues; that the same identical phosphates may be used indefinitely by animals whose food is deficient in this essential ingredient; and finally—which is still more significant—we have seen that these lime salts, of the deciduous teeth, are taken into the circulation at the period of life when these elements are the most imperatively demanded in the *growth* of the young animal.

If these positions have been maintained, we have an abundance of analogical evidence to conclude that the calcific elements of the deciduous teeth may be, and are, appropriated by the incomplete parts of the permanent teeth, or by any other tissue which may require these constituents as pabulum.

